# Structural basis of glycan specificity in neonate-specific bovine-human reassortant rotavirus

**DOI:** 10.1038/ncomms9346

**Published:** 2015-09-30

**Authors:** Liya Hu, Sasirekha Ramani, Rita Czako, Banumathi Sankaran, Ying Yu, David F. Smith, Richard D. Cummings, Mary K. Estes, B. V. Venkataram Prasad

**Affiliations:** 1 Verna and Marrs McLean Department of Biochemistry and Molecular Biology, Baylor College of Medicine, One Baylor Plaza, Houston, Texas 77030, USA; 2 Department of Molecular Virology and Microbiology, Baylor College of Medicine, Houston, Texas 77030, USA; 3 Berkeley Center for Structural Biology, Lawrence Berkeley National Laboratory, Berkeley, California 94720, USA; 4 Department of Biochemistry and the National Center for Functional Glycomics, Emory University School of Medicine, Atlanta, Georgia 30322, USA

## Abstract

Strain-dependent variation of glycan recognition during initial cell attachment of viruses is a critical determinant of host specificity, tissue-tropism and zoonosis. Rotaviruses (RVs), which cause life-threatening gastroenteritis in infants and children, display significant genotype-dependent variations in glycan recognition resulting from sequence alterations in the VP8* domain of the spike protein VP4. The structural basis of this genotype-dependent glycan specificity, particularly in human RVs, remains poorly understood. Here, from crystallographic studies, we show how genotypic variations configure a novel binding site in the VP8* of a neonate-specific bovine-human reassortant to uniquely recognize either type I or type II precursor glycans, and to restrict type II glycan binding in the bovine counterpart. Such a distinct glycan-binding site that allows differential recognition of the precursor glycans, which are developmentally regulated in the neonate gut and abundant in bovine and human milk provides a basis for age-restricted tropism and zoonotic transmission of G10P[11] rotaviruses.

## Abstract (web summary)

Rotaviruses cause life-threatening gastroenteritis in children and have been shown to have variations in glycan recognition from genetic alterations. Here, the authors use X-ray crystallography to indicate that these alterations can change the specificity of the virus from cows to infant humans.

## Graphical abstract

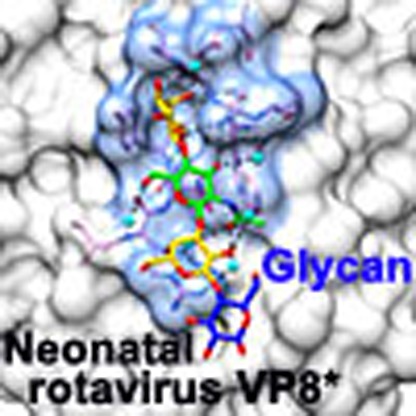

A critical step in the replication cycle of a virus is its initial attachment to cells mediated by the specific recognition of a cellular glycan, which often is a major determinant of host specificity^1^,^2^. For rotaviruses (RVs), which are the major causative agents of life-threatening gastroenteritis in children under the age of 5 years and result in 453,000 deaths worldwide annually, recognition of specific cellular glycans for initial cell attachment is mediated by the distally located VP8* domain of the spike protein VP4 (refs 3, 4). This protein along with the glycoprotein VP7 constitutes the outermost layer of the three concentric capsid layers that enclose the genome consisting of 11 double-stranded RNA segments^4^. RVs exhibit significant genetic and strain diversity. Similar to influenza viruses, point mutations, gene rearrangements and genetic reassortment between co-circulating strains, contribute to the expanding diversity of RVs^4^. Many of the human RV (HRV) strains are suggested to have originated from animal reservoirs through reassortment and inter-species transmission^5^,^6^. Although the currently licensed vaccines are effective in many countries, how effective they will remain in the face of such constantly expanding diversity is an open question^7^.

Based on differences in the genes encoding VP7 and the protease-sensitive VP4, RVs are classified into G (VP7) and P (VP4) genotypes^8^. Although structurally conserved with a galectin-like fold, sequence-wise VP8* is the least conserved among RV structural proteins contributing to a phylogeny consisting of 37 [P] genotypes^9^. Previously, it was thought that both ‘sialidase-sensitive’ animal RV (ARV) and ‘sialidase-insensitive’ HRV strains necessarily required interactions with sialoglycans for cell entry^10^. However, recent studies have shown that although recognition of sialoglycans is necessary for most ARVs^10^,^11^,^12^, HRVs exhibit genotype-dependent variations in glycan specificity^10^,^13^,^14^,^15^, which could be the basis for host tropism, host adaptation and zoonosis. More importantly, recent epidemiological studies^16^,^17^ indicate that the glycans mediate differences in genetic susceptibility to HRVs. However, our understanding of how genotypic changes in HRVs translate to variations in glycan recognition patterns to influence these factors is limited.

Among the HRVs, the naturally occurring bovine-human reassortant G10P[11] strains, bearing a VP4 of bovine origin, are known to preferentially infect neonates^18^,^19^. Although neonatal infections are largely asymptomatic, G10P[11] infections are also associated with severe gastrointestinal symptoms such as feed intolerance, necrotizing enterocolitis and abdominal distension in some settings^19^ An asymptomatic HRV strain 116E with a P[11] genotype has recently been licensed for use as a vaccine in India^20^. Microarray screening of 611 cellular non-sialylated and sialylated glycans and of a wide variety of human milk glycans have shown that VP8* of a G10P[11] HRV N155 strain recognizes glycans expressing Galβ-GlcNAc motifs with β1,**3** (type I) and β1,**4** (type II) linkages^21^,^22^,^23^,^24^. In contrast, the VP8* of a G10P[11] bovine RV (BRV) only binds to glycans with β1,**4** (type II) linkage^23^,^24^. The Galβ-GlcNAc motif is a precursor disaccharide for histo-blood group antigens (HBGAs), with β1,**3** linkage and β1,**4** linkages, referred to also as lacto-*N*-biose and *N*-acetyl-D-lactosamine, respectively. Precursor disaccharides containing the Galβ-GlcNAc motif are developmentally regulated in neonates and are the major building blocks of free glycans in human breast milk^25^. The synthesis of core precursor glycans is constitutive in many cell types. However, the addition of branches and terminal sugars are regulated in a tissue-specific manner during the postnatal period as part of normal development of the newborn^26^. Cell-based binding and infectivity assays indicate that glycans with Gal-GlcNAc motifs may serve as a potential cell receptor for the P[11] RVs^21^,^22^. Consistent with its restricted tropism to neonates, the human P[11] VP8* binds to saliva samples of neonates and infants but not to those of adults^22^. In addition, the infectivity of the vaccine P[11] HRV 116E was abrogated by the polyacrylamide (PAA)-conjugated poly*-N*-acetyl-D-lactosamine in human milk, and *Lycopersicon esculentum*-positive infant saliva^22^.

Although the structures of VP8* of several ARVs in complex with sialic acid (Sia) have been determined^11^,^12^,^27^, the only structure of a HRV VP8* with a bound glycan that is determined to date is that of P[14] VP8* in complex with A-type HBGA^14^. Here, we have focused on delineating the structural basis of the unique glycan specificity observed in a neonate-specific human strain and its bovine counterpart to gain insight into how the glycan specificity may contribute to age-restricted tropism and zoonosis. Our studies show how genotypic changes translate to novel structural elements in the neonate-specific P[11] VP8* to allow for its distinctive glycan specificity when compared with the VP8*s of other genotypes, and how subtle sequence alterations between the bovine and human P[11] VP8*s contribute to their differential glycan specificities.

## Results

### VP8* of P[11] HRV binds glycans with Galβ-GlcNAc motifs

**Figure 1:**
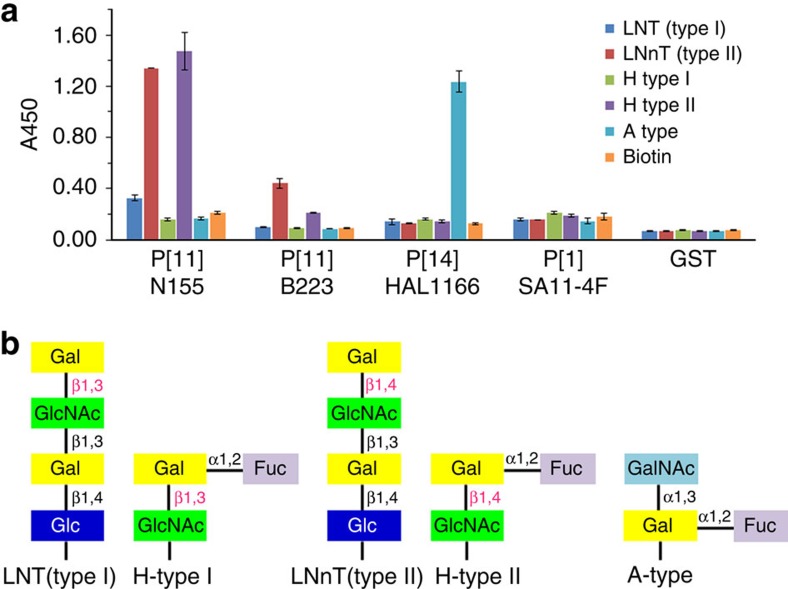
VP8* of neonate-specific P[11] RVs binds glycans with Galβ-GlcNAc motifs. (**a**) Binding of GST-tagged VP8*s to the PAA-conjugated and biotinylated glycans containing Galβ1,**3**GlcNAc or Galβ1,**4**GlcNAc motifs. As a control, VP8* of P[14] HRV HAL1166 that specifically binds to A-type HBGA trisaccharide was included. Each condition was tested with three replicates, and the entire experiment was reproduced. Each bar represents the mean A450 absorbance value. The error bars represent the standard deviation. (**b**) Schematic representation of the glycan structures. The glycan reagents were LNT tetrasaccharide (type I), LNnT tetrasaccharide (type II), H-type I trisaccharide, H-type II trisaccharide, A-type HBGA trisaccharide and biotin hydrazide reagent.

Previous glycan array studies have shown that the naturally occurring bovine-human reassortant G10P[11] HRV N155 binds to non-sialylated glycans containing the Galβ-GlcNAc precursor motifs with β1,**3** (type I) and β1,**4** (type II) linkages^23^,^24^ (also see Supplementary Table 1 for details). These studies show that although human P[11] VP8* recognizes glycans containing a common motif consisting of GlcNAcβ1,3-Galβ1,4-Glc with β1,**3** (type I) or β1,**4** (type II)-linked Gal at the non-reducing GlcNAc end, the bovine VP8* recognizes Galβ1,**4**-GlcNAcβ1,3-Galβ1,4-Glc (type II core). Consistent with these observations, for our crystallographic studies we used lacto-*N*-neotetraose (LNnT) and lacto-*N*-tetraose (LNT) tetrasaccharides with the sequences Galβ1,**4-**GlcNAcβ1,3-Galβ1,4-Glc and Gal β1,**3**-GlcNAcβ1,3-Galβ1,4-Glc, respectively, which represent these common motifs. Before our detailed crystallographic studies of the VP8* of P[11] HRV and that of the P[11] BRV, we validated their glycan specificities by carrying out ELISA binding assays using recombinant glutathione *S*-transferase (GST)-tagged VP8*s with PAA-conjugated and biotinylated type I (LNT) and type II (LNnT) glycans (Fig. 1). The binding of the VP8* of P[14] HRV HAL1166 to A-type HBGA was included as a positive control. Our results show that the VP8* of the neonate-specific P[11] HRV binds to either type I or II glycans, whereas the VP8* of BRV binds preferentially to type II glycans consistent with the previous studies (Fig. 1). As expected in the controls, the VP8* of P[14] HAL1166 showed specific binding to A-type HBGA, whereas the VP8*of P[1] ARV (SA11-4F strain, a simian RV strain binding to sialoglycans) did not bind to any of the non-sialylated glycans used in our studies.

### VP8* of P[11] HRV exhibits distinct structural changes

**Figure 2:**
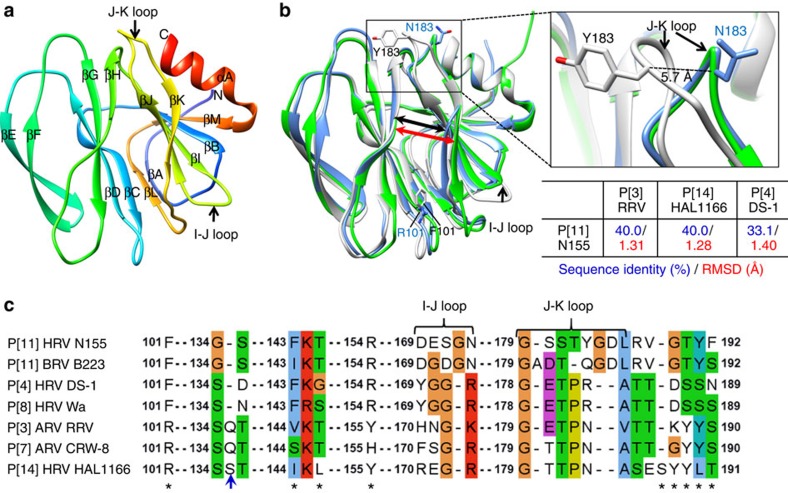
Crystal structure of P[11] human RV VP8*. (**a**) Ribbon representation of P[11] HRV N155 VP8* is coloured in rainbow and the strands are labelled with letters. (**b**) Superimposition of P[11] HRV N155 VP8* in grey with P[14] HRV HAL1166 VP8* (blue, PDB ID: 4DRV) and P[4] HRV DS-1 VP8* (green, PDB ID: 2AEN). The width of the cleft between two β-sheets in the P[14] VP8* is narrower (black double arrow) than in the P[11] VP8* and P[4] VP8* (red double arrow). The J-K loop that undergoes significant structural change is indicated by black arrow. The inlet shows the close-up view of the J-K loop region of all VP8*s. The distance between the Cα atom of Y183 in P[11] N155 VP8* and that of N183 in P[4] DS-1 VP8* is 5.7 Å. The RMSDs of the matching Cα atoms between the P[11] VP8* and other representative VP8*structures along with the percentage of sequence identity are shown. (**c**) The structure-based sequence alignment of VP8*s. The residues known to interact with Sia in animal rotaviruses (RRV and CRW-8), and the A-HBGA interacting residues in human rotavirus HAL1166 are indicated by *. The deletion at position 135 in VP8*s with a narrower cleft is labelled with a blue arrow.

The recognition of sialylated glycans by the VP8* of ARVs has been well characterized by the crystallographic studies of VP8* of P[3] and P[7] genotypes in complex with Sia^11^,^12^,^27^. For HRVs, although the crystal structures of VP8* of the commonly circulating P[4] (DS-1) and P[8] (Wa) strains have been determined^11^,^27^, the basis for their glycan recognition is not known because of the lack of their VP8* structures in complex with glycans. The only HRV structure in complex with a glycan is that of P[14] HRV (HAL1166) VP8* in complex with A-type HBGA. To understand how the genotypic sequence variations alter the structure and influence the glycan specificity in the neonate-specific P[11] HRV, we determined the crystal structure of VP8* of a P[11] human neonatal RV strain N155 isolated from a neonate with severe gastrointestinal disease in India^28^. As in other VP8* structures, the P[11] HRV VP8* adopts a galectin-like fold with two twisted antiparallel β-sheets consisting of strands A, L, C, D, G, H and M, B, I, J, K, respectively (Fig. 2a and Table 1). The two β-sheets are separated by a distinct cleft, which constitutes the glycan-binding site in the VP8* of ARVs and P[14] HRV. This cleft is noticeably wider in the P[11] HRV VP8* than that in the ARV VP8* structures or the P[14] VP8*; however, the width is similar to that observed in the VP8* of the globally dominant P[4] and P[8] HRVs (Fig. 2b).

Consistent with its distinct glycan-binding profile, the structure-based sequence alignment of representative VP8*s shows that the residues at the known Sia or A-HBGA-binding site are altered significantly in P[11] HRV N155 (Fig. 2c). For instance, R101 in the VP8*s of ARV and P[14] HRV, which is critical for glycan binding, is altered to a phenylalanine, whereas the conserved Y188 and Y189 are replaced by glycine and threonine, respectively, in the P[11] HRV VP8*. In addition to these amino acid changes, which may impact glycan specificity, there are significant structural alterations in the P[11] HRV VP8* structure, particularly in the I-J and J-K loops, which may also influence glycan specificity (Fig. 2b).

### P[11] HRV VP8* exhibits a novel glycan-binding site

**Figure 3:**
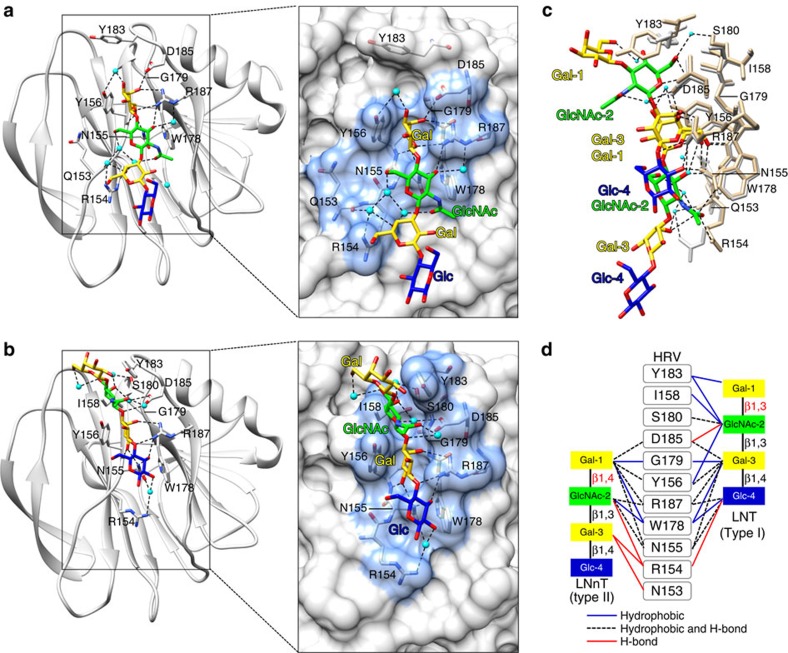
Interactions of LNnT and LNT with P[11] HRV N155 VP8*. Structure of P[11] HRV N155 VP8* in complex with LNnT (type II) (**a**) or LNT (type I) (**b**). The VP8* protein is shown in grey ribbon and surface (inlet figure) representations. The bound glycans are shown in stick representation with the β-D-Galactose (Gal) coloured yellow, the N-acetyl-D-glucosamine (GlcNAc) in green and the β-D-Glucose (Glc) in blue. Molecular interactions of LNnT and LNT with P[11] HRV N155 VP8* were analysed using LIGPLOT (also see Supplementary Fig. 2a,b). (**c**) Structural superimposition of the P[11] HRV VP8*(grey)/LNnT and P[11] HRV VP8* (tan)/LNT. The interacting residues is presented in stick model, and the glycan residues are labelled as in **a** and **b**. (**d**) The schematic diagram of the interaction between HRV P[11] VP8* with both glycans.

In the VP8* structures of ARV and of P[14] HRV, the Sia and A-type HBGA, respectively, bind to a very similar region in the narrow cleft. Subtle sequence alterations within the same structural framework in these VP8*s permit switching from Sia in the ARVs to A-type HBGA in the P[14] HRV. To understand the structural basis for the glycan specificity in the neonate-specific P[11] HRV VP8*, how the observed sequence and structural alterations configures the glycan-binding site, and how it accommodates the Galβ-GlcNAc motif with either β1,**3** or β1,**4** linkages, we first determined the structure of P[11] HRV VP8* with LNnT. The co-crystals diffracted to 1.28 Å. The density for the bound LNnT was clearly observed in the simulated annealing omit map allowing unambiguous modelling of the ligand (Supplementary Fig. 1a). The bound glycan spans almost the entire length of the cleft stabilized by a network of hydrogen-bonding interactions and several hydrophobic interactions (Fig. 3a and Supplementary Fig. 2a). This binding site is quite distinct from that observed in ARV or P[14] VP8*s, in which the glycan binds at one corner of the cleft region (Supplementary Fig. 3). Residues N155, Y156, W178, G179 and D185 of the P[11] VP8* engage in hydrophobic interactions with the terminal Galβ1,**4-**GlcNAc moieties, which are further stabilized by direct hydrogen bond interactions with N155 and R187, and water-mediated hydrogen bonds involving Y156. The Gal3 moiety of the glycan, which is linked to GlcNAc via β1,3 linkage, interacts with Q153 through water-mediated hydrogen-bonding interactions (Fig. 3a and Supplementary Fig. 2a). The Glc4 moiety, at the reducing end of the glycan, projects away from the VP8* without making any contacts. A similar set of interactions as seen with Galβ1,**4-**GlcNAc are also possible with H-type II HBGA, which has the same type II precursor but with an additional Fuc linked to Gal via α1,2 linkage providing a structural explanation for the observed binding of this glycan in both glycan array and ELISA experiments. Based on the position of the O_2_ atom of the Gal residue in the structure, the Fuc moiety of HBGA would project away into the solvent without causing any steric clashes.

### Glycan-binding site accommodates type I and type II glycans

To investigate how P[11] HRV VP8* can also bind to Galβ-GlcNAc motif with β1,**3** linkage, we determined the structure of P[11] HRV VP8* in complex with LNT to a resolution of 1.5 Å (Fig. 3b and Supplementary Fig. 1b). In comparison with type II LNnT, the type I LNT is shifted exactly by one disaccharide unit towards the J-K loop such that Galβ1,4Glc at the reducing end of the LNT occupies the same position as the terminal Galβ1,**4-**GlcNAc of LNnT involving the same set of hydrophobic and hydrogen-bonding interactions (Fig. 3c,d). The residues N155, Y156, W178, G179 and D185, which provide hydrophobic interactions with the terminal Galβ1,**4-**GlcNAc moieties of LNnT, now interact with the reducing end Galβ1,4-Glc of LNT sharing a common set of hydrogen bond and hydrophobic interactions. The non-reducing terminal Galβ1,**3-**GlcNAc of LNT, because of the shift, makes a new set of hydrophobic interactions involving I158, S180 and Y183 (Supplementary Fig. 2b). Based on computational analysis, using AutoDock 4.2 (ref. 29), the shared interactions involving β1,**4** galactose contributes more significantly than the other residues towards the overall binding energy, suggesting that the specificity for these two ligands is largely provided by β1,**4**-linked galactose residue. Although Galβ1,4-Glc of LNT contributes −5.27 kcal mol^−1^ out of a total binding energy of −7.36 kcal mol^−1^, Galβ1,4-GlcNAc of LNnT contributes −6.05 kcal mol^−1^ out of −6.21 kcal mol^−1^.

### P[11] VP8* exhibits linkage-specific conformational changes

**Figure 4:**
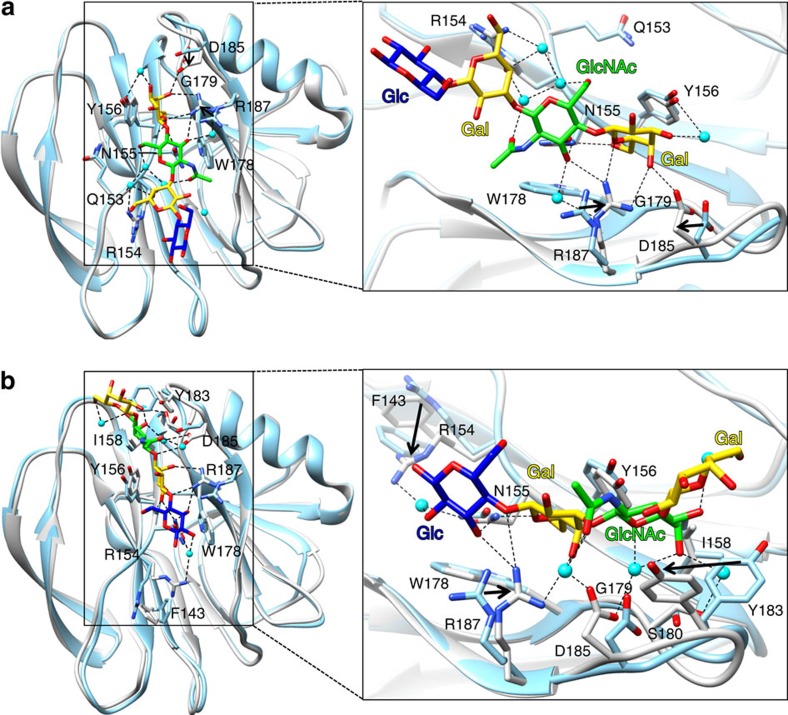
Conformational changes of P[11] HRV VP8* upon binding to glycans. (**a**) Structural comparison of the P[11] HRV apo structure (light blue) with the liganded P[11] HRV VP8* (grey) with LNnT (type II), with the inlet showing the close-up view of the structural changes in a different orientation. (**b**) Structural comparison of the P[11] HRV apo structure (light blue) with the liganded P[11] HRV VP8* (grey) with LNT (type I), with the inlet showing the close-up view of the structural changes as in **a**. The interacting residues are shown in stick model, and the glycan residues are labelled as in Fig. 3. Changes in the sidechain orientations are indicated by black arrows.

To examine whether P[11] VP8* undergoes differential conformational changes upon binding to LNnT with a type II linkage and LNT with a type I linkage, we overlaid the unliganded and the LNnT or LNT-bound P[11] HRV VP8* structures. The superposition of the unliganded and LNnT-bound P[11] HRV VP8*structures, which matched well with an root mean square deviation (rmsd) of 0.65 Å, showed that two conserved residues R187 and R154 in the co-crystal structure changed their sidechain orientations to hydrogen bond with Gal1 and Gal3 moieties, respectively (Fig. 4a). Comparison of the unliganded and LNT-bound P[11] VP8* structures also showed that ligand binding induces significant changes. The R154 side chain that pairs with the aromatic side chain of F143 via a cation–π interaction in the unliganded structure, upon LNT binding, swings around to interact with the Gal3 residue, through a water-mediated hydrogen bond, and the side chain of F143 rotates accordingly to maintain the cation–π interaction (Fig. 4b). The side chain of S180 that hydrogen bonds with the main chain NH of Y183 in the unliganded structure reorients upon LNT binding to interact with the GlcNAc moiety through a water-mediated hydrogen bond interaction. A significant change that is observed in the LNT-bound structure is with respect to Y183 in the J-K loop. The side chain of Y183 is rotated to create a hydrophobic pocket to stabilize the terminal Galβ1,**3**-GlcNAc of LNT. Without this side chain reorientation, Y183 would clash with the GlcNAc residue (Fig. 4b).

### Zoonotic bovine P[11] VP8* exhibits structural differences

**Figure 5:**
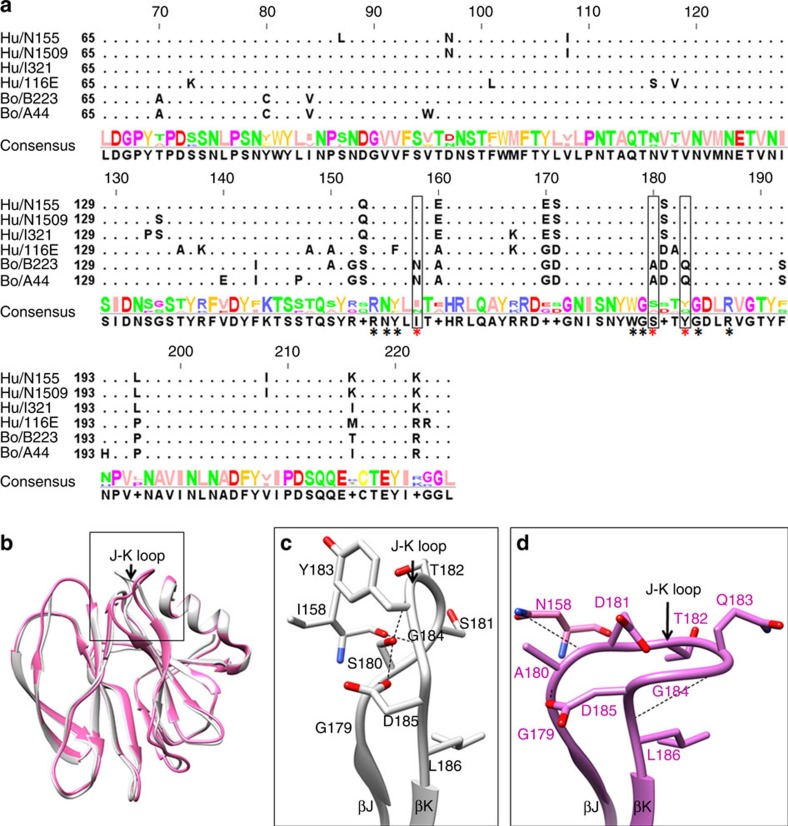
Crystal structure of P[11] bovine RV (BRV) VP8*. (**a**) Sequence alignment of P[11] VP8*s of various representative P[11] human RV (HRV) and BRV strains. The consensus amino acids are coloured using Clustal protein colour scheme in Jalview^50^. The black * indicates the residues recognizing both LNnT and LNT, and the red * indicates those only bind LNT in P[11] HRV N155. The key amino acid changes between HRV and BRV strains are marked by black boxes. (**b**) Structural overlay of the structure of P[11] HRV N155 VP8* coloured in grey with that of the P[11] BRV B223 VP8* in pink. The significant structural variations of the J-K loops are indicated by a black box. (**c**) The J-K loop of P[11] HRV VP8* shows the hydrogen bond interactions between the amino acids leading the loop projecting the strand H. (**d**) The J-K loop of P[11] BRV VP8* presented in the same orientation and scale as in **c**. All the residues present in the J-K loop are shown as stick model.

Despite belonging to the same genotype, the human and bovine P[11] VP8* show differences in their glycan specificity. Although P[11] HRV recognizes the Galβ-GlcNAc motif of either β1,**3** or β1,**4** linkage, P[11] BRV binds only to the β1,**4** motif. Sequence comparison of human and bovine P[11] VP8*s shows 86% amino-acid identity. The residues interacting with the type II glycan in the P[11] HRV VP8* are conserved in the BRV VP8*, indicating that the bovine VP8* can also bind glycans with the β1,**4** motif using the same binding site (Fig. 5a). However, there are several significant amino acid changes from human to bovine P[11], including S180A, Y183Q, I158N and F192S. To investigate whether the sequence variations within the VP8*s of the same genotype lead to any structural changes, and how they account for differential glycan binding, we determined the crystal structure of VP8* from a P[11] BRV strain B223 at 2.2 Å resolution (Fig. 5b). Comparison of the human and bovine P[11] VP8* structures shows that despite the overall structural similarity (RMSD=0.66 Å, Fig. 5b), the J-K loop in the bovine VP8*, which has several sequence alterations as noted above, undergoes significant structural change. In the bovine VP8*, this loop with a different set of inter-residue hydrogen bonds projects in an opposite direction compared with that of the human VP8* (Figs 5c,d). The backbone conformational angles in this loop from residues G179 to G184 are also significantly altered.

### Structural changes confer differential glycan specificity

**Figure 6:**
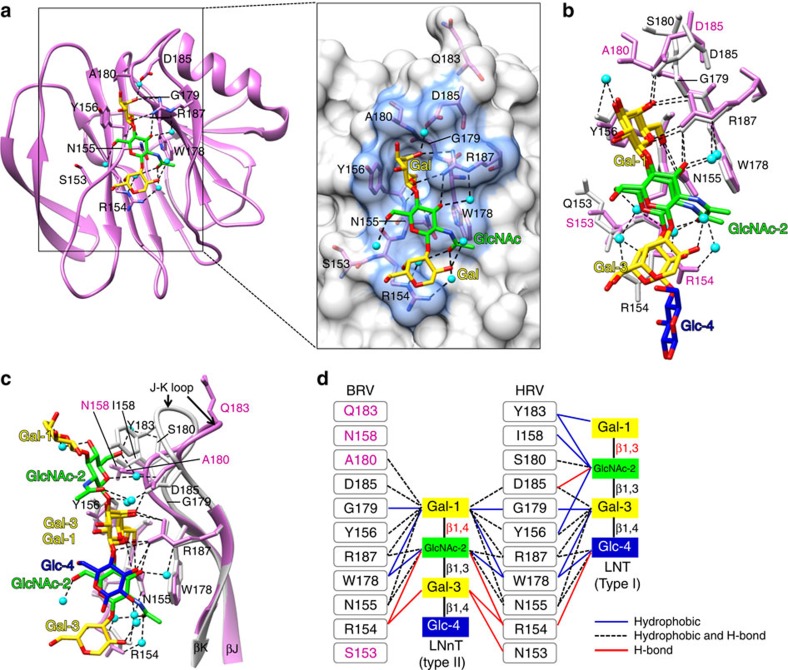
Interactions between LNnT and P[11] BRV VP8*. (**a**) Structure of P[11] BRV VP8*(pink) in complex with LNnT (type II). The VP8* protein is shown in grey ribbon and surface (inlet figure) representations. The glycans are shown in stick representation with the β-D-Galactose (Gal) coloured yellow, the N-acetyl-D-glucosamine (GlcNAc) in green and the β-D-Glucose (Glc) in blue. Molecular interactions of LNnT with BRV N155 P[11] VP8* were analysed using LIGPLOT (also see Supplementary Fig. 2c). (**b**) Superimposition of the structure of P[11] BRV VP8*(pink) or P[11] HRV VP8*(grey) in complex with LNnT (type II). The interacting residues are shown in stick model. The interacting residues is presented in stick model, and the glycan residues are labelled as in **a**. (**c**) Structural overlay of P[11] BRV VP8*(pink)/LNnT complex with P[11] HRV VP8*(grey)/LNT complex. (**d**) The schematic diagram of glycan recognition in both HRV and BRV VP8*s shows how the amino acid changes in P[11] BRV lead to loss of binding to type I glycan LNT, and how both VP8*s recognize type II glycan LNnT in a similar manner.

To test our hypothesis that both human and bovine P[11] VP8*s use the same binding site to interact with type II glycan, we co-crystallized bovine P[11] VP8* with LNnT (Fig. 6a and Supplementary Fig. 1c). The crystals, with four molecules in the asymmetric unit, diffracted to 1.46 Å. The crystal structure shows that the Galβ1,**4-**GlcNAc moieties of LNnT at the non-reducing end interact at the same binding site as in the human P[11] VP8* with similar hydrophobic interactions involving the conserved N155, Y156, W178, G179 and D185 and hydrogen bond interactions involving the conserved R154 and R187 (Fig. 6b and Supplementary Fig. 2c). The electron density for the Glc moiety at the reducing end, however, is not well defined in any of the four molecules in the asymmetric unit because of the lack of engagement by the protein and was not modelled in the final structure (Supplementary Fig. 1c). Similar to P[11] HRV VP8*, bovine VP8* also undergoes conformational changes upon binding to LNnT including reorientation of the side chains of R154 and R187 (Supplementary Fig. 4). In the ligand-bound bovine P[11] VP8* structure, in all the four molecules of the asymmetric unit, the conformation of the J-K loop remains the same as in the apo structure, despite the space group difference, indicating that the different conformation of the J-K loop from that observed in the human P[11] VP8* is mainly due to the sequence variations and not the ligand binding or crystal contacts (Supplementary Figs 4 and 5).

To understand the structural basis for the preferential binding of type II glycans in the case of bovine P[11] VP8*, in contrast to human VP8*, which binds both type I and II, we superimposed the crystal structure of human P[11] VP8* in complex with LNT with the LNnT-bound bovine P[11] VP8* structure (Fig. 6c). Comparative analysis clearly shows that the amino acid changes S180A, Y183Q, I158N along with the altered conformation of the J-K loop in the bovine VP8* result in the loss of the binding pocket for type I Galβ1,**3-**GlcNAc motif (Fig. 6c and Fig. 6d). These residues in the human P[11] VP8* make substantial interactions with this motif in LNT. The residue Y183 in the human VP8* that is involved in a stabilizing hydrophobic stacking interaction with the type I glycan (Fig. 3b) is replaced by Q183 in the bovine VP8*, which in the structure is shifted 5.3 Å away because of the conformational change in the J-K loop (Fig. 5c). S180, which interacts with β1,**3**-linked GlcNAc of LNT through stabilizing hydrophobic and hydrogen-bonding interactions, is replaced by A180, which based on the superposition of the structures clashes with this moiety. In addition, I158 that is involved in a hydrophobic interaction with GlcNAc in the human VP8* is replaced by a hydrophilic residue N158. Consistent with the structural observation, co-crystallization of P[11] BRV B223 VP8* with LNT using similar concentrations as the LNnT did not show any density for the bound LNT, indicating that LNT cannot bind to B223 VP8*. Remarkably, all these residues are conserved in the respective human and bovine P[11] strains, suggesting zoonotically related bovine and human P[11] strains display similar differential glycan specificities (Fig. 5a).

## Discussion

Our crystallographic analyses provide the structural basis to explain the unique glycan specificity exhibited by a neonate-specific bovine-human reassortant RV strain and its parental bovine strain, each belonging to the P[11] genotype. The results strongly impact our understanding of the age-restricted tropism and inter-species transmission of RVs. We have shown that P[11] VP8* exhibits a novel glycan-binding site that adapts to a differential recognition of precursor glycans, which are the core elements of HBGA and human/bovine milk glycans. Our studies show although the glycan-binding site in the VP8* of the parental bovine strain is configured to specifically recognize the type II glycans, the binding site is reconfigured to recognize either type I or type II glycans in the neonate-specific strain because of subtle sequence variations.

Despite the same galectin-like polypeptide fold, as found in other VP8*s, P[11] VP8* display significant differences particularly in the glycan-binding site. To date, the glycan-binding site in the VP8* of several ARV strains, which recognize sialoglycans, has been structurally characterized^11^,^12^,^30^. The only available structure of the VP8* of a HRV strain with bound glycan is that of the P[14] HRV strain, which recognizes non-sialo A-type HBGA^14^. The overlapping glycan-binding sites in these VP8*s are localized to one corner of the cleft that separates the two twisted antiparallel sheets^14^. A distinguishing feature of the P[11] VP8* is that glycan binding occurs in a previously uncharacterized binding pocket and the binding cleft is noticeably wider. This wider cleft is significantly more expansive and can accommodate two or three sugar residues of the bound glycan. The width of the cleft in the P[11] VP8* is, more similar to that observed in the VP8* structures of the globally dominant P[4] and P[8] HRV strains despite significant sequence alterations. Based on the currently available VP8* structures, a structure-based analysis of the VP8* sequences indicates that the wider cleft strongly correlates with a deletion at position 135, and a significant change at position 101, which is a conserved arginine residue contributing to glycan interactions in the VP8*s with a narrow cleft^27^. Based on this correlation, it can be predicted that the VP8* of another HRV strain belonging to P[6] genotype will also exhibit a wider cleft. All these HRVs have a zoonotic origin. It is possible that a preponderance of particular types of glycans in the hosts, to which RVs can adapt with appropriate sequence changes, may provide a selective evolutionary pressure for such segregation.

Structural comparison of animal and human P[14] VP8*s, each with a narrow cleft in complex with Sia and A-type HBGA, respectively, demonstrates how subtle sequence changes within overlapping glycan-binding sites switch the specificity from Sia to A-type HBGA. Similarly, it is possible that the glycan-binding site in the VP8* of P[4], P[8] and possibly in P[6], which have a wider cleft, overlaps with the new glycan-binding site discovered in the P[11] VP8*, with sequence changes in accordance with their glycan specificity. Although the structures of P[4], and P[8] VP8* with glycans are yet to be determined, recent studies using nuclear magnetic resonance, supported by infectivity assays, have shown that VP8* of these strains exhibit more complex interactions likely involving multiple sugar residues^13^. These studies show that P[4] (DS-1) and P[6] (RV-3) HRV strains recognize A-type HBGA as does P[14] HRV. However, unlike the A-type binding in the P[14] VP8* where the interaction occurs predominantly with the Gal and GlcNAc residues, the interactions in P[4] and P[6] strains additionally involve the α1,2-linked fucose of the HBGA. Similar studies, however, indicate that glycan specificity is significantly altered in the Wa-strain that represents a globally dominant P[8] genotype^10^. This strain does not interact with HBGA-related glycans but predominantly recognizes the sialoglycan ganglioside GM1a^10^. It is possible that a wider cleft in the VP8*s of HRVs provides a more adaptable platform for diversification of strain-dependent glycan specificity allowing sustained viral evolution among the human population. As new data on glycan partners for commonly circulating HRVs continue to be identified, infectivity assays will also allow us to determine the functional significance of VP8*–glycan interactions in the strains that cause severe gastrointestinal disease.

Specific recognition of the core features of glycans by the bovine- and the neonate-specific P[11] RV strains, as demonstrated by glycan microarrays^21^,^22^,^23^, infectivity assays^22^ and now substantiated by our crystallographic analyses, is rather unique. The origin of this glycan specificity may arise from the fact that these glycans are developmentally regulated in neonates and they are abundantly present in human and bovine milk. Glycans in the gut are derived from diet and from host mucosal secretions. For a newborn, the primary sources of dietary glycans are those present in breast milk or infant formula. As human milk oligosaccharides (HMOs) beyond lactose are not readily digested by human enzymes, these glycans may have evolved as natural prebiotics to support the growth of gut microbiota in the newborn^31^. HMOs contain both type I and II glycans, predominantly the type I (ref. 32), and these may be processed by the beneficial gut bacteria into constituent precursor glycans necessary for structuring the neonate gut^25^. Although little is known about the roles of bovine milk glycans or bovine microbiota, it is likely that bovine milk, which contains exclusively or predominantly type II glycans^33^, serves the same purpose. Thus, the BRV strain may have achieved specificity for the type II glycans in the microenvironment of the bovine gut, and with a few sequence changes, may have evolved to cross the species barrier to recognize both type II and type I glycans, which may be present abundantly in the human gut epithelium particularly during the developmental stages. Genotypic alterations allowing neonate-specific P[11] strains to recognize both type I and type II receptors may indeed be the basis for zoonotic transmission. Additional infectivity studies are required to determine the biological significance of P[11] VP8* binding to the specific type I and type II glycans described in this study.

The role of HMOs acting as decoy receptors in protecting the neonates from pathogens is well documented^25^. One conundrum is why HMOs do not protect neonates from P[11] RV infection, because P[11] infections have been observed in breastfed neonates, although the severity of these infections in breastfed neonates compared with those who are not breastfed is not known. The ability of neonate-specific P[11] RV to subvert the protective effects of human milk may arise from the unusual ability of this virus, as we observed, to recognize the tandem repeats of the type I and II precursor glycans of the HMO. It is interesting that galectins, which are abundant in epithelial cells, especially galectins-1 and -3 (refs 34, 35), also bind to tandem repeats of type I or II glycans; such interactions in conjunction with glycan binding by P[11] VP8* with a similar polypeptide fold to the carbohydrate domain in galectins might alter signalling pathways to facilitate viral entry. Recently, crystal structures of human galectin-3 bound to LNT and LNnT, which are components of glycosphingolipids, have been reported^36^. Within the complexes, both LNT and LNnT adopt an extended linear conformation very similar to that observed in the P[11] VP8*, suggesting a manner in which the core LNT and LNnT structures bound by VP8* could engage both galectins and P[11] VP8. Other studies have suggested that galectins may be exploited for transmission and replication of viruses, such as influenza viruses, HIV and Nepah viruses^37^,^38^. Clearly, the possible role of galectins and their ligands in affecting the cell entry and replication of RVs needs further studies.

## Methods

### Protein purification

Recombinant N-terminal GST-tagged VP8*s of G10P[11] human neonatal RVs N155 and the G10P[11] BRV B223 were expressed in *Escherichia coli* BL21 (DE3) cells (Novagen) and purified via Glutathione Sepharose 4 Fast Flow (GE Healthcare)^14^,^21^. The GST tag was cleaved by the protease thrombin before rebinding the protein mixtures onto a Glutathione Sepharose column to remove the GST and the uncleaved fusion protein, leaving Gly-Ser at the N terminus. The VP8* was then filtered and further purified by size exclusion chromatography on a Superdex-75 (GE Healthcare) column with 10 mM Tris, pH 7.4, 100 mM NaCl, 1 mM dithiothreitol. The concentration of the purified VP8* was determined by measuring absorbance at 280 nm and using an absorption coefficient of 37,360 M^−1^ cm^−1^ (N155) and 34,505 M^−1^ cm^−1^ (B223) calculated using ProtParam on the ExPASy Server^39^.

### Enzyme-linked immunosorbent assay

The binding of purified VP8* to different glycans was confirmed by ELISA. All reagents were diluted in 0.1 M sodium phosphate buffer with 0.25% fatty acid-free bovine serum albumin (Sigma-Aldrich) and all assay volumes were 100 μl. Neutravidin-coated 96-well plates (Pierce Thermo Fisher Scientific) were coated for 2 h at room temperature with 2.5 μg ml^−1^ of the following glycans, in triplicate: H type 1-PAA-biotin, H type 2-PAA-biotin, A antigen-(PAA)-biotin (GlycoTech) or with biotinylated LNT tetrasaccharide or biotinylated LNnT tetrasaccharide (GlycoTech). Negative control wells were coated with biotin hydrazide reagent (Pierce Thermo Fisher Scientific). Purified, 100 μg ml^−1^ GST-tagged VP8* was incubated with immobilized glycans for 2 h at 4 °C. Plates were washed three times with assay buffer and bound GST-VP8* was detected by horseradish peroxidase-conjugated anti-GST (0.1 μg ml^−1^, GenScript, Cat. No. A00130-40). Colour was developed by adding tetramethylbenzidine peroxidase liquid substrate (Pierce Thermo Fisher Scientific). The reaction was stopped after 10 min by adding 1 M phosphoric acid. Absorbance at 450 nm was measured using a SpectraMax M5 plate reader (Molecular Devices). The cutoff value for binding was calculated using the mean of the negative control wells plus 3 s.d. of the negative control optical density (OD) values. An OD value above this cutoff was considered as positive binding, whereas an OD value equal to or below the cutoff was indicative of lack of binding.

### Crystallization

Crystallization screens for P[11] VP8* at concentrations of 16–20 mg ml^−1^ were carried out by hanging-drop vapour diffusion using the Mosquito crystallization robot (TTP LabTech) and visualized using Rock Imager (Formulatrix) at 20 °C. The P[11] VP8*s of HRV N155 and BRV B223 were crystallized alone or co-crystallized with 10–15 mM LNnT or LNT (obtained from CFG or purchased from Dextra Labs). The unliganded N155 was crystallized under the condition with 20% PEG8000, 0.1 M CHES (N-Cyclohexyl-2-aminoethanesulfonic acid), pH 9.0. The N155 VP8* was co-crystallized with LNT under the condition with 0.94 M sodium citrate, 0.1 M imidazole, pH 8.0. The N155 VP8*/LNnT co-crystals were obtained from screening condition with 0.2 M NaCl, 0.1 M Imidazole, pH 8.0, 1.0 M potassium/sodium tartrate. The unliganded B223 VP8* was crystallized under condition with 0.1 M Tris, 1.6 M ammonium sulfate, pH 8.0, and the B223 VP8*/LNnT was crystallized with 0.1 M phosphate citrate, pH 4.2, 1.6 M sodium dihydrogen phosphate, 0.4 M di-potassium hydrogen phosphate. The crystals were flash frozen in liquid nitrogen.

### Data collection and processing

Diffraction data for the unliganded and liganded P[11] VP8* crystals were collected on 5.0.1 beamline at Advanced Light Source, Lawrence Berkeley National Laboratory. These data were processed with IMOSFLM as implemented in the CCP4 suite^40^. Space groups were confirmed using POINTLESS^41^. The HAL1166 VP8* native structure (PDB ID: 4DRR) was used as a search model for molecular replacement using PHASER^42^. From the molecular replacement solution, considering the high-resolution data obtained for all the crystals, automated model building and solvent addition were carried out using ARP/wARP^43^. The atomic model including the side chain atoms obtained from ARP/wARP was then subjected to iterative cycles of refinement using PHENIX^44^ and further model building using COOT^45^ based on the difference maps. The LNnT and LNT moieties were generated using the SWEET2 package^46^ of the Glycosciences.de server (http://www.glycosciences.de) and modelled into the electron density using COOT^45^. The density and stereochemistry of the glycans, and the conformational changes in the VP8* were validated by computing simulated annealing omit maps using PHENIX^44^ and the CARP package^47^. Data collection and refinement statistics following final refinement cycle are given in Table 1 (see also representative 2Fo-Fc density maps in Supplementary Fig. 6). Ligand interactions were analysed using LIGPLOT^48^. The structural alignments and calculations of RMSD were carried out using the Chimera^49^. AutoDock 4.2 was used to compute the binding energies of the ligands^29^. Figures were prepared by using Chimera.

## Additional information

**How to cite this article:** Hu, L. *et al*. Structural basis of glycan specificity in neonate-specific bovine-human reassortant rotavirus. *Nat. Commun.* 6:8346 doi: 10.1038/ncomms9346 (2015).

## Supplementary Material

Supplementary Information Supplementary Figures 1-6, Supplementary Table and Supplementary Reference.

## Figures and Tables

**Table 1:** Crystallographic statistics of P[11] HRV VP8* and P[11] BRV VP8* structures.

	P[11] HRV VP8*	P[11] HRV VP8*	P[11] HRV VP8*	P[11] BRV VP8*	P[11] BRV VP8*
Ligand	None	LNnT	LNT	None	LNnT
Protein Data Bank ID	4YFW	4YG0	4YFZ	4YG3	4YG6
					
*Data collection*					
Space group	P2_1_	P2_1_2_1_2_1_	P2_1_2_1_2_1_	P2_1_	P2_1_
					
*Cell dimensions*					
*a*, *b*, *c* (Å)	35.53, 71.94, 58.05	32.21, 64.05, 70.92	54.96, 55.28, 56.57	28.81, 64.73, 36.75	36.20, 140.28, 54.60
*α, β, γ* (°)	90, 90.02, 90	90, 90, 90	90, 90, 90	90, 114.87, 90	90, 90.78, 90
Wavelength (Å)	0.9774	0.9774	0.9774	0.9774	0.9774
Resolution (Å)	36.53–1.66 (1.75–1.66)	31.02–1.28 (1.35–1.28)	32.10–1.50 (1.58–1.50)	30.0–2.28 (2.32–2.28)	36.2–1.46 (1.54–1.46)
*R*_merge_ (%)	8.0 (27.8)	6.5 (28.3)	12.5 (24.5)	11.2 (22.3)	7.4 (33.3)
Number of molecules in the asymmetric unit	2	1	1	1	4
Completeness (%)	98.1 (97.2)	92.2 (99.8)	99.9 (100.0)	99.2 (99.6)	99.0 (99.5)
Redundancy	3.9 (3.9)	8.1 (7.1)	8.0 (8.3)	3.7 (3.6)	4.0 (4.2)
					
*Refinement*					
Resolution (Å)	36.53–1.66	31.02–1.28	32.10–1.50	29.64–2.29	36.20–1.46
No. of reflections	65,091	66,379	53,290	5,503	182,017
*R*_work_/*R*_free_ (%)	22.2/25.5	17.2/19.9	17.6/20.8	18.7/24.2	17.7/19.7
					
*Average B factor (Å*^*2*^)					
Protein	10.64	9.85	11.30	22.82	10.52
Ligand		18.94	12.67		13.57
Water	21.11	21.66	23.35	25.17	27.51
					
*R.M.S. deviations*					
Bond length (Å)	0.008	0.007	0.007	0.003	0.007
Bond angles(°)	1.130	1.102	1.123	0.665	1.100

BRV, bovine rotavirus; HRV, human rotavirus; LNT, lacto-*N*-tetraose; LNnT, lacto-*N*-neotetraose.

Numbers in parenthesis correspond to highest resolution shell.
