# Supplementary material for: Structural basis of glycan specificity in neonate-specific bovine-human reassortant rotavirus
Source: Nat Commun. 2015 Sep 30;6:8346. doi: 10.1038/ncomms9346 (PMC4589887; doi:10.1038/ncomms9346)
Supplement: Supplementary Information — Supplementary Figures 1-6, Supplementary Table and Supplementary Reference. [file ncomms9346-s1.pdf]

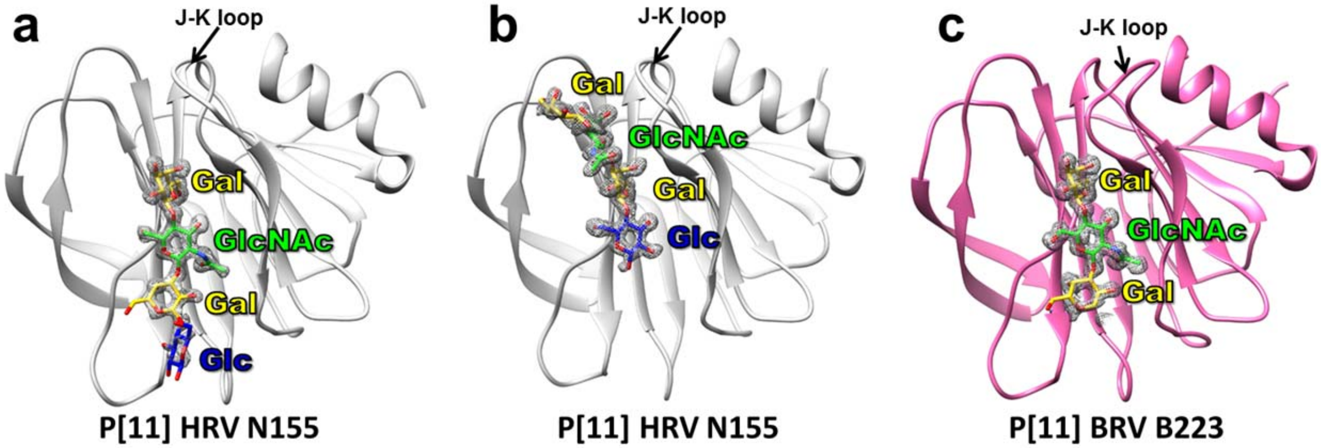

Supplementary Figure 1. Electron density map of the glycan receptors in the three P[11] VP8*/glycan complex structures. **(a).** The P[11] HRV N155 VP8* in complex with type II glycan LNnT. **(b).** The P[11] HRV N155 VP8* in complex with type I glycan LNT. **(c).** The P[11] BRV B223 VP8* in complex with type II glycan LNnT. The panels show the unbiased simulated omit electron density maps (Fo-Fc) for the bound glycan receptors contoured at 3.0 sigma. The Fo-Fc maps are generated by FFT program in CCP4 software. In all panels the glycans are shown in stick representation with the β-D-Galactose (Gal) colored yellow, the N-acetyl-D-glucosamine (GlcNAc) in green and the β-D-Glucose (Glc) in blue.

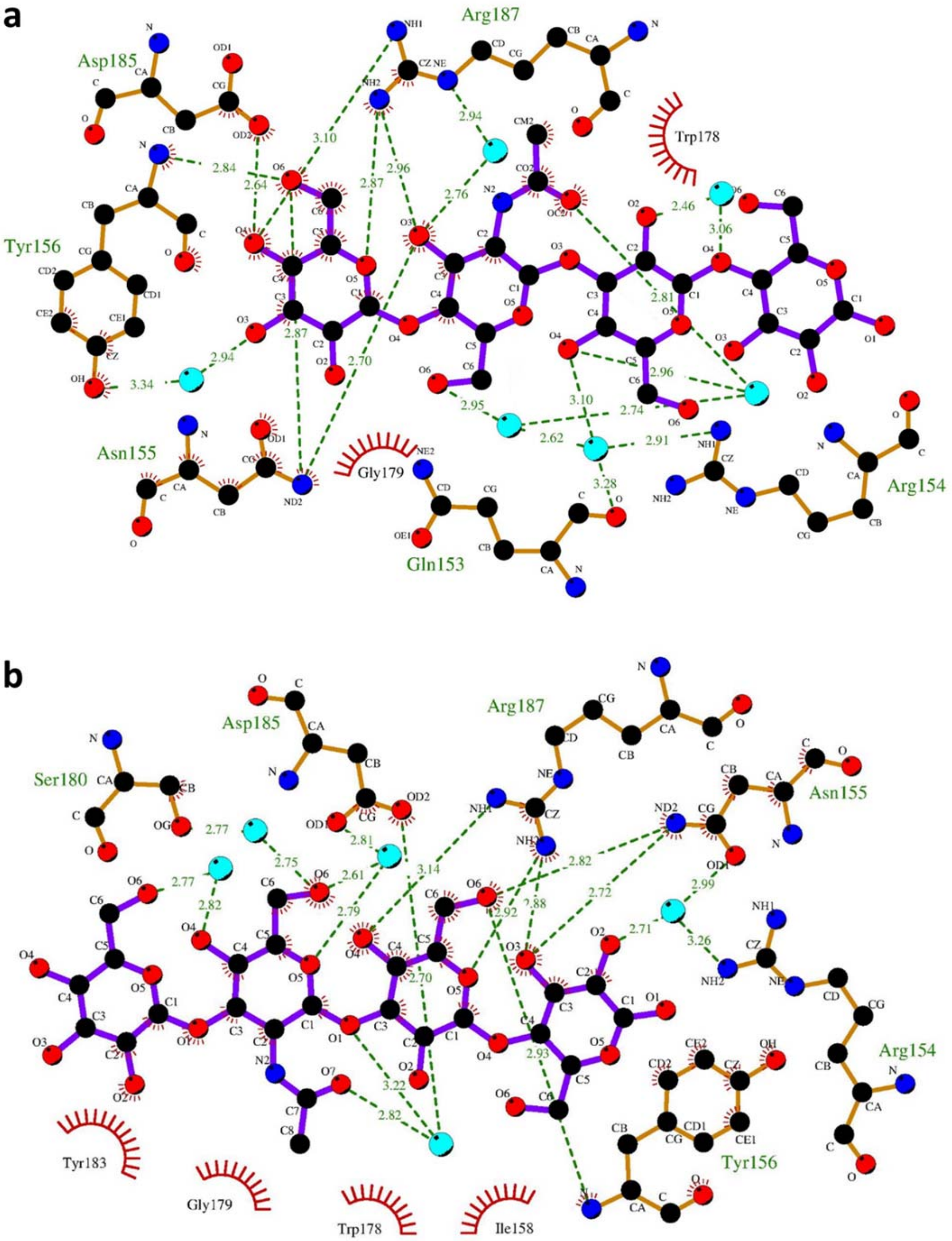
a
Arg187
Asp185
Trp178
Tyr156
Asn155
Gly179
Gln153
Arg154
b
Arg187
Asp185
Ser180
Asn155
Arg154
Tyr156
Tyr183
Gly179
Trp178
Ile158

c

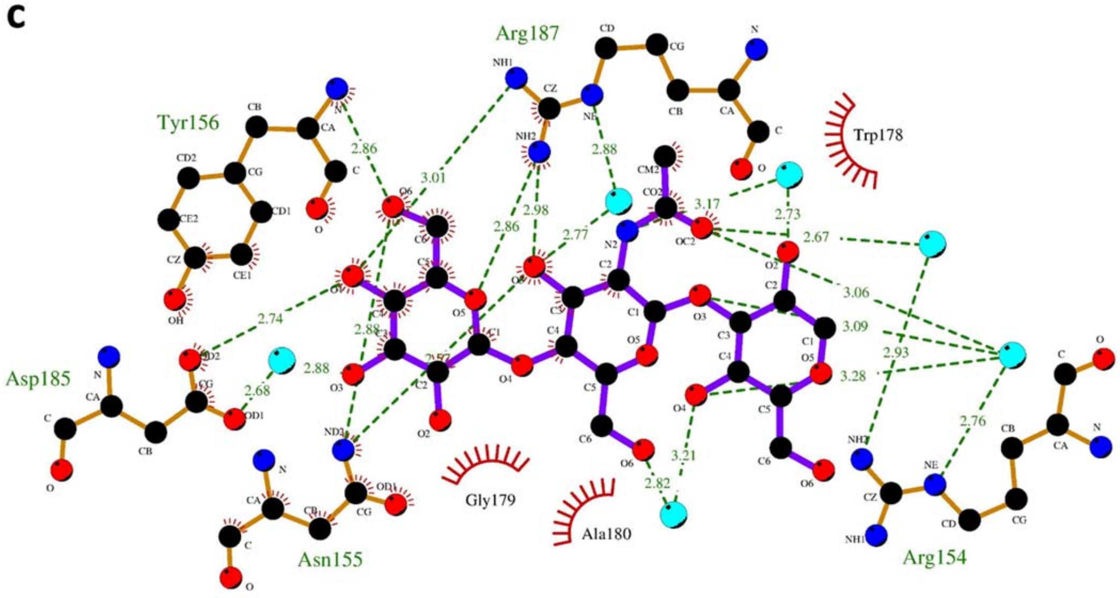

Supplementary Figure 2. Detailed VP8* ligand interactions as determined using LIGPLOT. (a) Human RV N155 interactions with type II glycan LNnT; **(b)** Human RV N155 interactions with type I glycan LNT; **(c)** Bovine RV B223 interactions with type II glycan LNnT.

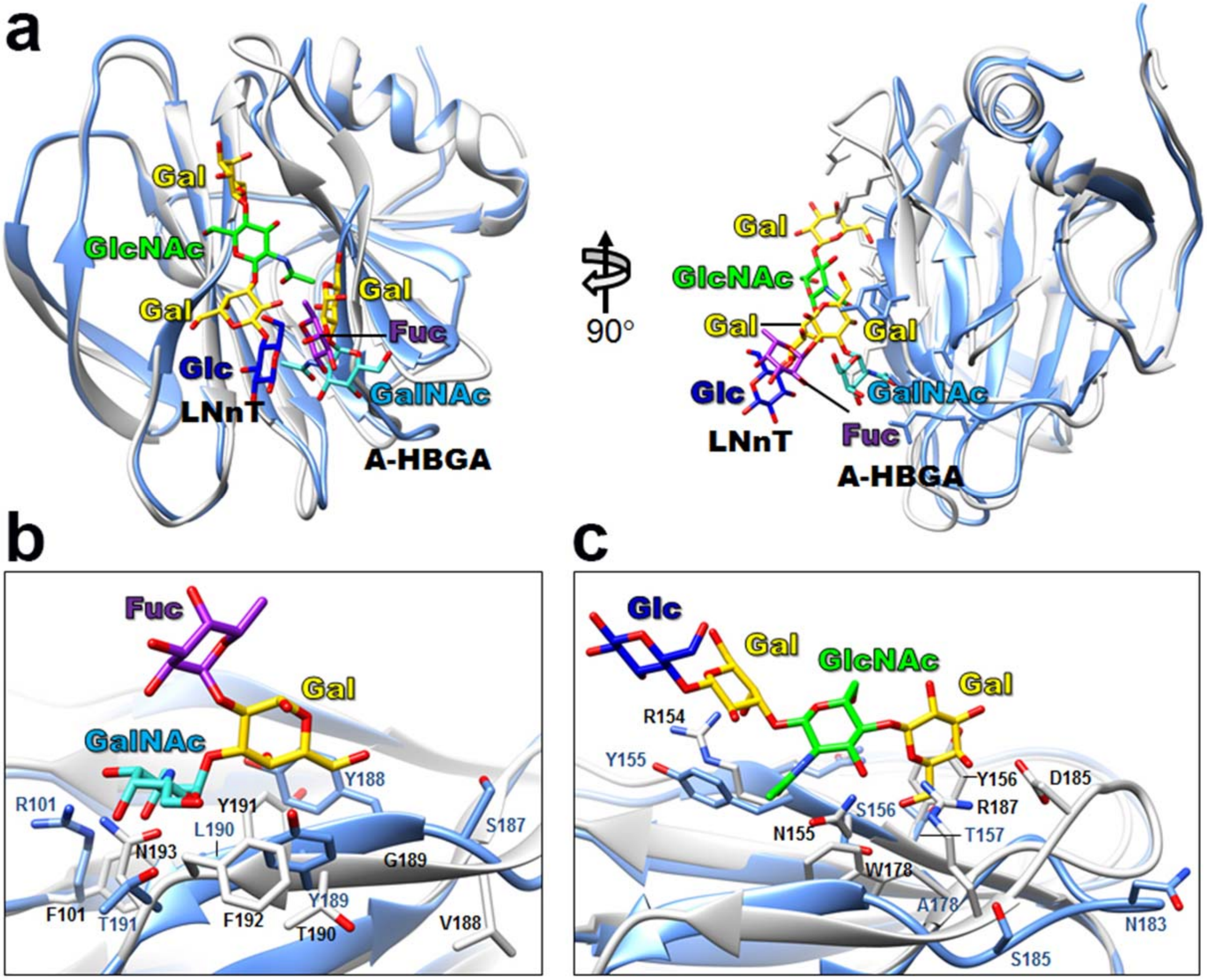

Supplementary Figure 3. Comparison of the glycan binding sites of P[14] HRV HAL1166 and P[11] HRV N155. (a). Structure overlay of the P[14] HRV HAL1166/A-type HBGA and P[11] HRV N155/LNnT. The P[14] HRV VP8* and P[11] HRV VP8* are shown as blue and grey ribbons, respectively. The bound glycans are represented with stick model and labeled individually. **(b).**Close-up view of the A-type HBGA binding site in P[14] HRV VP8* showing the amino acid changes in P[11] HRV VP8* abolish binding to A-HBGA at this region. **(c).**Close-up view of the LNnT binding site in P[11] HRV VP8* shows the sequence and structure difference at this site between two HRVs.

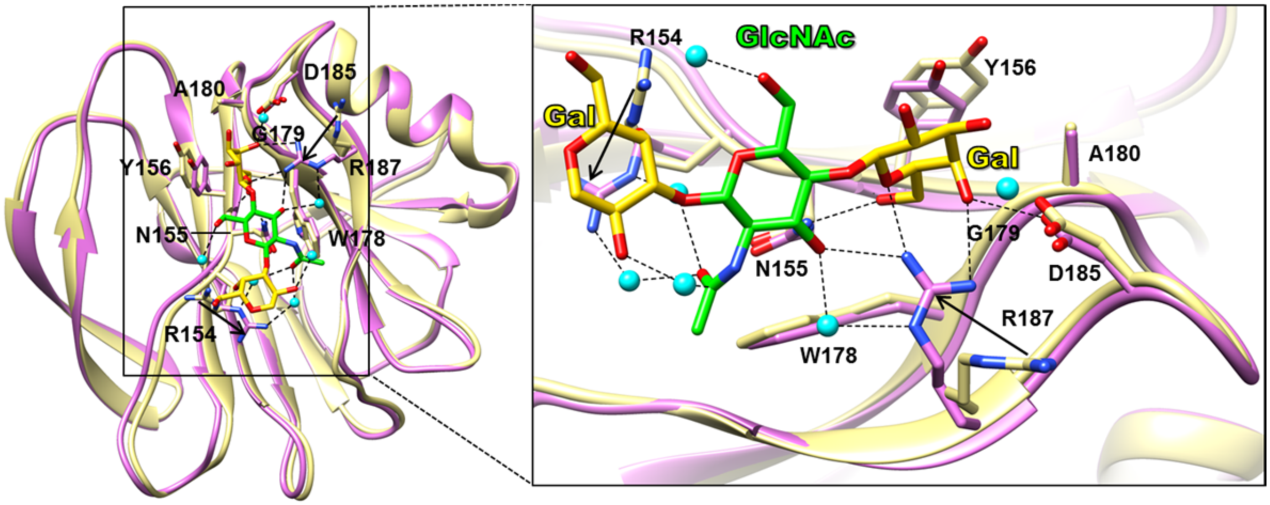

Supplementary Figure 4. Conformational changes of P[11] BRV VP8* upon binding to glycans. (a). Structural comparison of the P[11] BRV apo structure (khaki) with the liganded BRV P[11] VP8* (pink) with LNnT (type II), with the inlet showing the close-up view of the structural changes in a different orientation. The interacting residues are shown in stick model, and the glycan residues are labeled as in **Fig 6**.

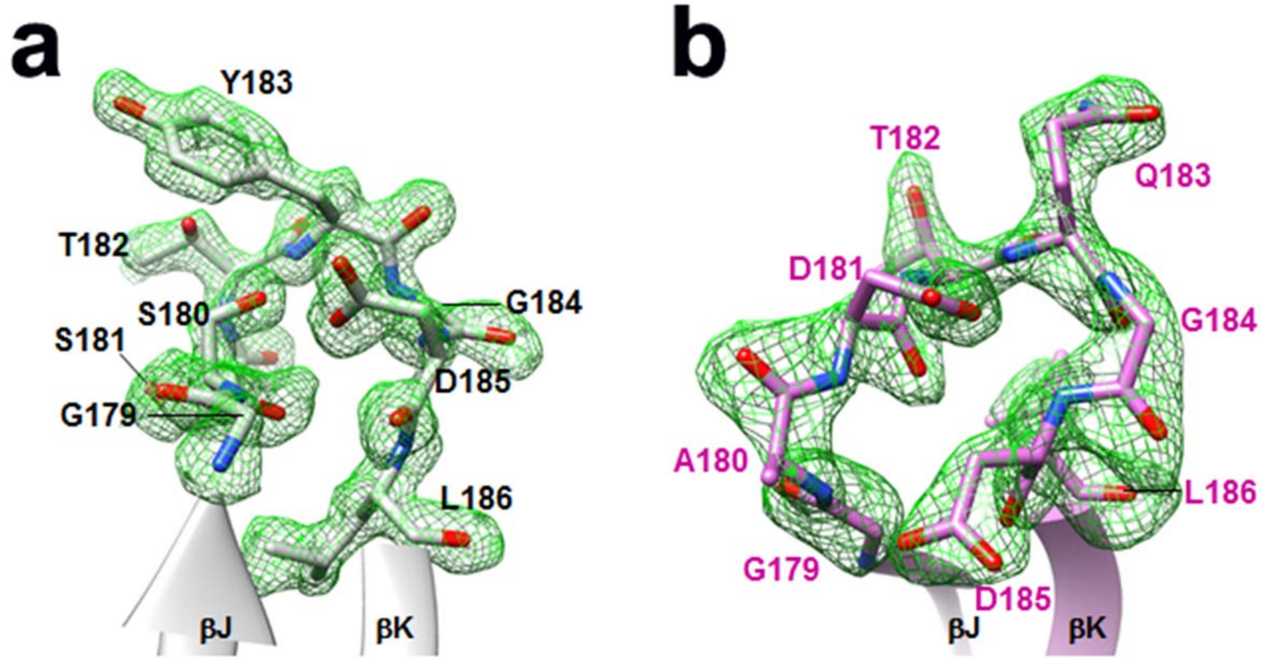

Supplementary Figure 5. Conformational changes in J-K loop between human and bovine VP8*. The unbiased simulated omit electron density maps (Fo-Fc) of the J-K loop in VP8* apo structures of the P[11] HRV **(a)** or BRV **(b)** is shown with green mesh and contoured at 3.0 sigma. The electron density of this loop in other structures has similar quality.

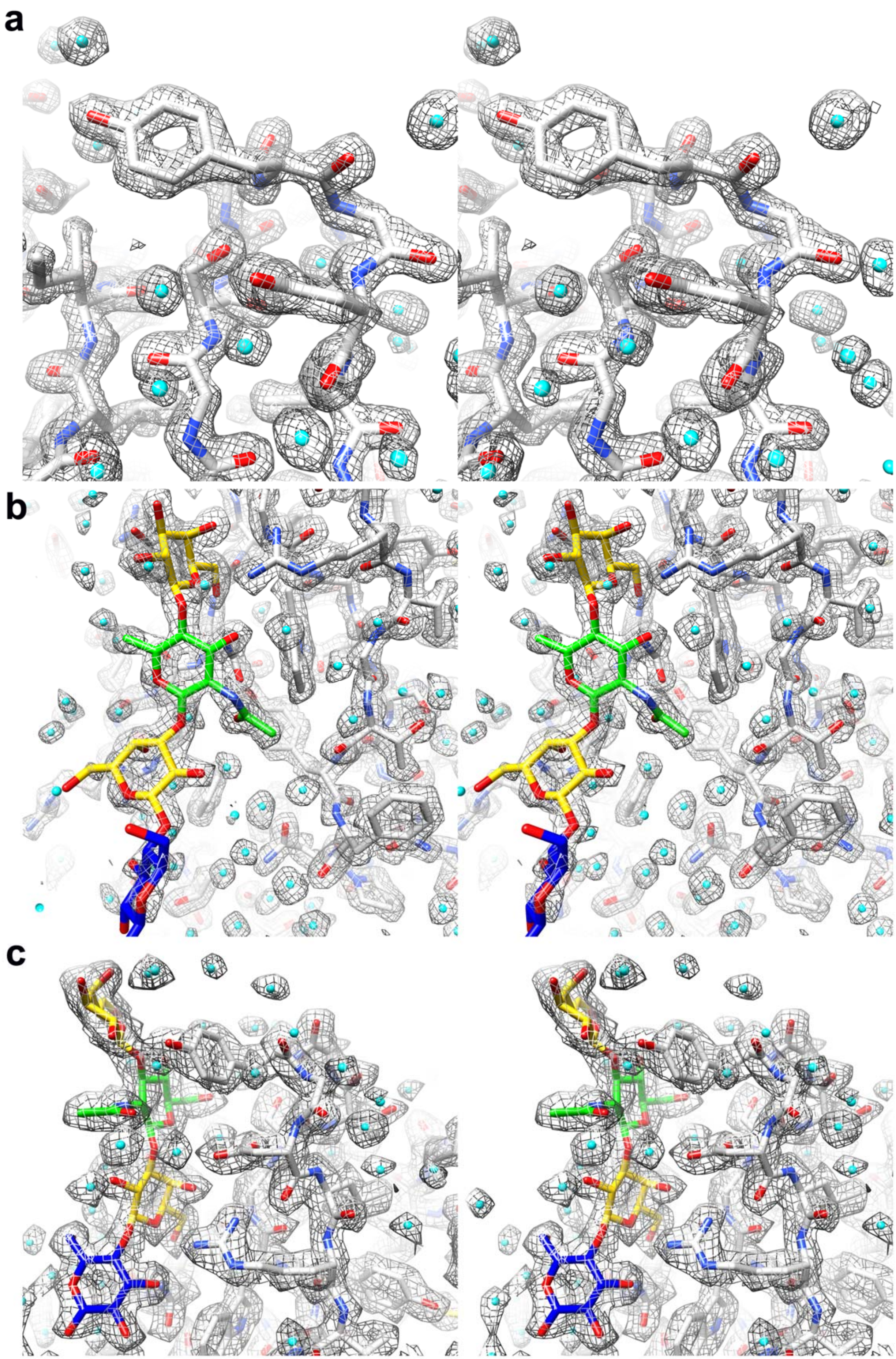
a
b
c

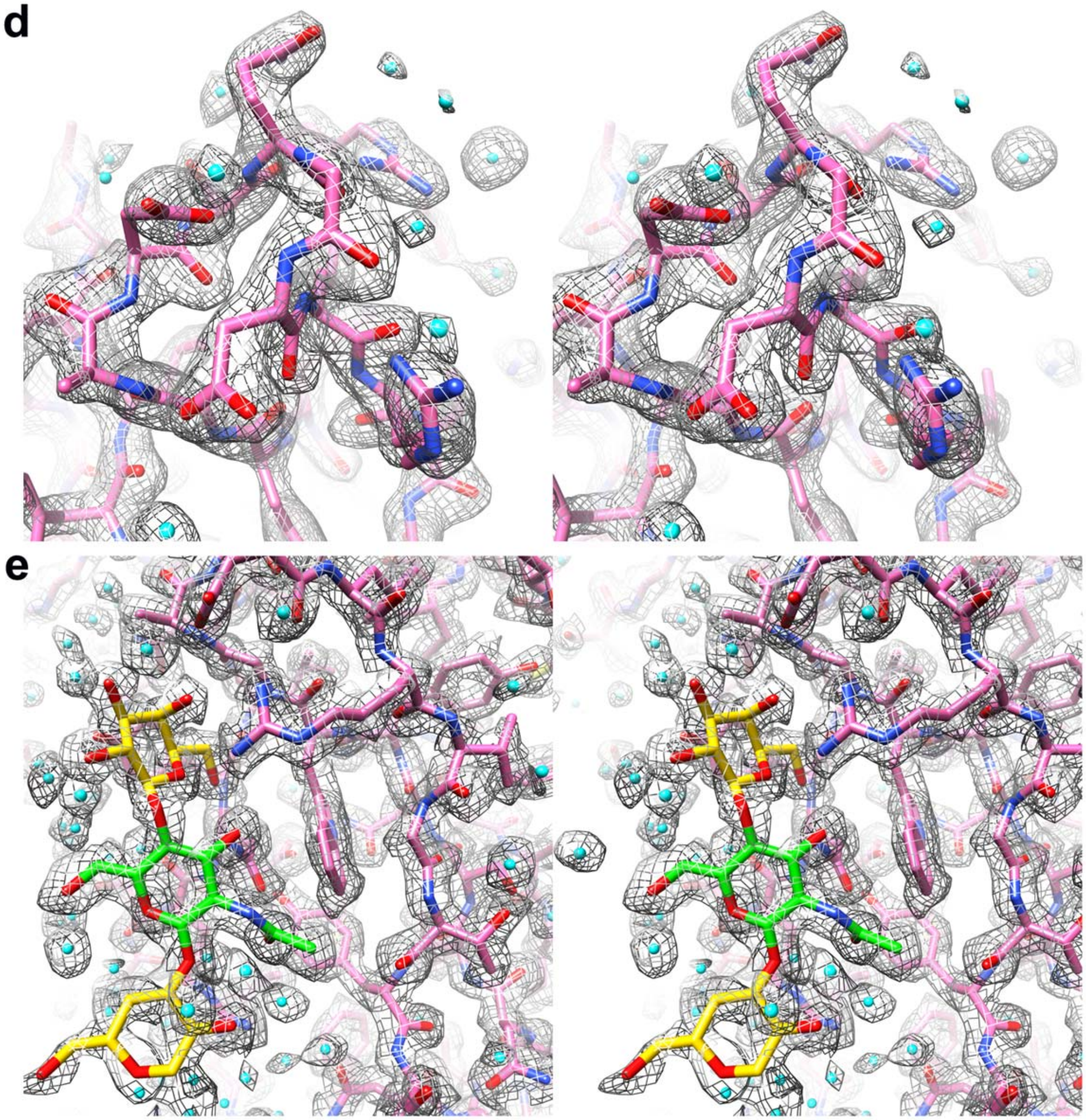

Supplementary Figure 6. Representative electron density maps of the structures reported here.

Representative stereo image of 2Fo-Fc maps contoured at 1.0σ (grey mesh) in the structures of P[11] HRV VP8*, (**a**) apo, (**b**) in complex with LNnT, and (**c**) in complex with LNT; and the structures of P[11] BRV VP8*, (**d**) apo and (**e**) in complex with LNnT,

Supplementary Table S1.

Glycan Array results for GST-P[11] HRV N155 VP8* protein tested at a concentration of 20μg/ml on CFG glycan microarray v5.0. The array is comprised of 611 glycans printed on a microscope slide, and the protein is applied to the array in 70 μL under a coverslip and incubated for 1 hr. at room temperature. The slide is then washed with buffer to remove unbound protein and excess buffer is removed using a slide centrifuge. GST-P[11] HRV N115 VP8* bound to glycans on the array is then detected using the same procedure with 70 μL of 5 μg/ml anti-GST antibody labeled with Alexa488. The excess antibody is removed by washing and the slide is dried. The Fluorescence is measured using a Fluorescence slide scanner (ProScanArray, PerkinElmer) and the relative strength of binding of the protein to the glycan is presented as relative fluorescence units (RFU). Standard deviation and % coefficient of variation (%CV) from the mean of 6 replicates of each glycan printed on the array is provided, and the top 92 glycans are arranged in descending order of RFU where values >200 are considered significantly different from background based on a z-score. Glycans 163 and 75 (highlighted in green) are extended Type II and H-Type II determinants, respectively[1]. Glycans 74 (H type II) and 164 (LnNT) and 166 (LnNT), highlighted in blue, are shorter versions of H-Type II and Type II determinants, respectively[1]. Glycans 581, 582 and 588 (highlighted in orange) represent poly LacNac (repeating Galβ1-4GlcNAcβ1-3Galβ1-4GlcNAc), which also are a multivalent representation of the LNnT determinant in a polysaccharide context. Glycans 572, 573, 552, 383 contain type I precursor sequence.

Chart Number	P[11] HRV N155 VP8 (20ug/ml) CFG# 2456 Slide#:14539 Alexa488 CMZ	Average RFU	StDev	% CV
581	GlcNAcb1-3Galb1-4GlcNAcb1-3Galb1-4GlcNAcb1-3Galb1-4GlcNAcb1-3Galb1-4GlcNAcb1-2Mana1-6(GlcNAcb1-3Galb1-4GlcNAcb1-3Galb1-4GlcNAcb1-3Galb1-4GlcNAcb1-3Galb1-4GlcNAcb1-2Mana1-3)Manb1-4GlcNAcb1-4(Fuca1-6)GlcNAcb-Sp19	48865	682	1
582	Galb1-4GlcNAcb1-3Galb1-4GlcNAcb1-3Galb1-4GlcNAcb1-3Galb1-4GlcNAcb1-3Galb1-4GlcNAcb1-2Mana1-6(Galb1-4GlcNAcb1-3Galb1-4GlcNAcb1-3Galb1-4GlcNAcb1-3Galb1-4GlcNAcb1-3Galb1-4GlcNAcb1-2Mana1-3)Manb1-4GlcNAcb1-4(Fuca1-6)GlcNAcb-Sp19	47360	3270	7
588	GlcNAcb1-3Galb1-4GlcNAcb1-3Galb1-4GlcNAcb1-3Galb1-4GlcNAcb1-3Galb1-4GlcNAcb1-6(GlcNAcb1-3Galb1-4GlcNAcb1-3Galb1-4GlcNAcb1-3Galb1-4GlcNAcb1-3Galb1-4GlcNAb1-2)Mana1-6(GlcNAcb1-3Galb1-4GlcNAcb1-3Galb1-4GlcNAcb1-3Galb1-4GlcNAcb1-3Galb1-4GlcNAcb1-2Mana1-3)Manb	45709	1585	3
551	Galb1-4GlcNAcb1-3Galb1-4GlcNAcb1-3Galb1-4GlcNAcb1-3Galb1-4GlcNAcb1-2Mana1-6(Galb1-4GlcNAcb1-3Galb1-4GlcNAcb1-3Galb1-4GlcNAcb1-3Galb1-4GlcNAcb1-2Mana1-3)Manb1-4GlcNAcb1-4GlcNAcb-Sp25	45690	2503	5
565	GlcNAcb1-3Galb1-4GlcNAcb1-3Galb1-4GlcNAcb1-3Galb1-4GlcNAcb1-3Galb1-4GlcNAcb1-2Mana1-6(GlcNAcb1-3Galb1-4GlcNAcb1-3Galb1-4GlcNAcb1-3Galb1-4GlcNAcb1-3Galb1-4GlcNAcb1-2Mana1-3)Manb1-4GlcNAcb1-4GlcNAcb-Sp25	45239	2874	6
589	Galb1-4GlcNAcb1-3Galb1-4GlcNAcb1-3Galb1-4GlcNAcb1-3Galb1-4GlcNAcb1-3Galb1-4GlcNAcb1-6(Galb1-4GlcNAcb1-3Galb1-4GlcNAcb1-3Galb1-4GlcNAcb1-3Galb1-4GlcNAcb1-3Galb1-4GlcNAb1-2)Mana1-6(Galb1-4GlcNAcb1-3Galb1-4GlcNAcb1-3Galb1-4GlcNAcb1-3Galb1-4GlcNAcb1-3Galb1-4G	44971	1074	2
587	Galb1-4GlcNAcb1-3Galb1-4GlcNAcb1-3Galb1-4GlcNAcb1-3Galb1-4GlcNAcb1-6(Galb1-4GlcNAcb1-3Galb1-4GlcNAcb1-3Galb1-4GlcNAcb1-3Galb1-4GlcNAb1-2)Mana1-6(Galb1-4GlcNAcb1-3Galb1-4GlcNAcb1-3Galb1-4GlcNAcb1-3Galb1-4GlcNAcb1-2Mana1-3)Manb1-4GlcNAcb1-4(Fuca1-6)GlcNAcb-	44619	1347	3
566	Galb1-4GlcNAcb1-3Galb1-4GlcNAcb1-3Galb1-4GlcNAcb1-3Galb1-4GlcNAcb1-3Galb1-4GlcNAcb1-2Mana1-6(Galb1-4GlcNAcb1-3Galb1-4GlcNAcb1-3Galb1-4GlcNAcb1-3Galb1-4GlcNAcb1-3Galb1-4GlcNAcb1-2Mana1-3)Manb1-4GlcNAcb1-4GlcNAcb-Sp25	44550	2247	5
549	Galb1-4GlcNAcb1-3Galb1-4GlcNAcb1-3Galb1-4GlcNAcb1-2Mana1-6(Galb1-4GlcNAcb1-3Galb1-4GlcNAcb1-3Galb1-4GlcNAcb1-2Mana1-3)Manb1-4GlcNAcb1-4GlcNAcb-Sp24	43442	1720	4
569	Galb1-4GlcNAcb1-3Galb1-4GlcNAcb1-3Galb1-4GlcNAcb1-3Galb1-4GlcNAcb1-3Galb1-4GlcNAcb1-3Galb1-4GlcNAcb1-2Mana1-6(Galb1-4GlcNAcb1-3Galb1-4GlcNAcb1-3Galb1-4GlcNAcb1-3Galb1-4GlcNAcb1-3Galb1-4GlcNAcb1-3Galb1-4GlcNAcb1-2Mana1-3)Manb1-4GlcNAcb1-4GlcNAcb-Sp25	43215	1244	3
580	Galb1-4GlcNAcb1-3Galb1-4GlcNAcb1-3Galb1-4GlcNAcb1-3Galb1-4GlcNAcb1-2Mana1-6(Galb1-4GlcNAcb1-3Galb1-4GlcNAcb1-3Galb1-4GlcNAcb1-3Galb1-4GlcNAcb1-2Mana1-3)Manb1-4GlcNAcb1-4(Fuca1-6)GlcNAcb-Sp24	41276	1667	4
550	GlcNAcb1-3Galb1-4GlcNAcb1-3Galb1-4GlcNAcb1-3Galb1-4GlcNAcb1-2Mana1-6(GlcNAcb1-3Galb1-4GlcNAcb1-3Galb1-4GlcNAcb1-3Galb1-4GlcNAcb1-2Mana1-3)Manb1-4GlcNAcb1-4GlcNAcb-Sp25	40250	1194	3
579	GlcNAcb1-3Galb1-4GlcNAcb1-3Galb1-4GlcNAcb1-3Galb1-4GlcNAcb1-2Mana1-6(GlcNAcb1-3Galb1-4GlcNAcb1-3Galb1-4GlcNAcb1-3Galb1-4GlcNAcb1-2Mana1-3)Manb1-4GlcNAcb1-4(Fuca1-6)GlcNAcb-Sp24	40036	1573	4
591	Galb1-4GlcNAcb1-3Galb1-4GlcNAcb1-6(Galb1-3)GalNAca-Sp14	39066	5168	13
578	Galb1-4GlcNAcb1-3Galb1-4GlcNAcb1-3Galb1-4GlcNAcb1-2Mana1-	38063	2481	7

	6(Galb1-4GlcNAcb1-3Galb1-4GlcNAcb1-3Galb1-4GlcNAcb1-2Mana1-3)Manb1-4GlcNAcb1-4(Fuca1-6)GlcNAcb-Sp24			
586	GlcNAcb1-3Galb1-4GlcNAcb1-3Galb1-4GlcNAcb1-3Galb1-4GlcNAcb1-6(GlcNAcb1-3Galb1-4GlcNAcb1-3Galb1-4GlcNAcb1-3Galb1-4GlcNAb1-2)Mana1-6(GlcNAcb1-3Galb1-4GlcNAcb1-3Galb1-4GlcNAcb1-3Galb1-4GlcNAcb1-2Mana1-3)Manb1-4GlcNAcb1-4(Fuca1-6)GlcNAcb-Sp24	34991	1060	3
585	Galb1-4GlcNAcb1-3Galb1-4GlcNAcb1-3Galb1-4GlcNAcb1-6(Galb1-4GlcNAcb1-3Galb1-4GlcNAcb1-3Galb1-4GlcNAb1-2)Mana1-6(Galb1-4GlcNAcb1-3Galb1-4GlcNAcb1-3Galb1-4GlcNAcb1-2Mana1-3)Manb1-4GlcNAcb1-4(Fuca1-6)GlcNAcb-Sp24	25439	1695	7
601	Neu5Aca2-3Galb1-4GlcNAcb1-3Galb1-4GlcNAcb1-6(Galb1-3)GalNAca-Sp14	23734	1861	8
602	Neu5Aca2-6Galb1-4GlcNAcb1-3Galb1-4GlcNAcb1-6(Galb1-3)GalNAca-Sp14	21709	1367	6
547	GlcNAcb1-3Galb1-4GlcNAcb1-3Galb1-4GlcNAcb1-2Mana1-6(GlcNAcb1-3Galb1-4GlcNAcb1-3Galb1-4GlcNAcb1-2Mana1-3)Manb1-4GlcNAcb1-4GlcNAcb-Sp25	18615	165	1
577	GlcNAcb1-3Galb1-4GlcNAcb1-3Galb1-4GlcNAcb1-2Mana1-6(GlcNAcb1-3Galb1-4GlcNAcb1-3Galb1-4GlcNAcb1-2Mana1-3)Manb1-4GlcNAcb1-4(Fuca1-6)GlcNAcb-Sp24	15965	1137	7
572	Galb1-3GlcNAcb1-3Galb1-4GlcNAcb1-3Galb1-4GlcNAcb1-6(Galb1-3GlcNAcb1-3Galb1-4GlcNAcb1-3Galb1-4GlcNAb1-2)Mana1-6(Galb1-3GlcNAcb1-3Galb1-4GlcNAcb1-3Galb1-4GlcNAcb1-2Mana1-3)Manb1-4GlcNAcb1-4(Fuca1-6)GlcNAcb-Sp24	14782	541	4
576	Galb1-4GlcNAcb1-3Galb1-4GlcNAcb1-2Mana1-6(Galb1-4GlcNAcb1-3Galb1-4GlcNAcb1-2Mana1-3)Manb1-4GlcNAcb1-4(Fuca1-6)GlcNAcb-Sp24	13397	837	6
552	Galb1-3GlcNAcb1-3Galb1-4GlcNAcb1-2Mana1-6(Galb1-3GlcNAcb1-3Galb1-4GlcNAcb1-2Mana1-3)Manb1-4GlcNAcb1-4GlcNAc-Sp25	10720	119	1
75	Fuca1-2Galb1-4GlcNAcb1-3Galb1-4GlcNAcb1-3Galb1-4GlcNAcb-Sp0 **(H-type II)**	9880	491	5
545	Fuca1-2Galb1-4GlcNAcb1-3Galb1-4GlcNAcb1-2Mana1-6(Fuca1-2Galb1-4GlcNAcb1-3Galb1-4GlcNAcb1-2Mana1-3)Manb1-4GlcNAcb1-4GlcNAcb-Sp24	9445	291	3
543	Galb1-4GlcNAcb1-3Galb1-4GlcNAcb1-2Mana1-6(Galb1-4GlcNAcb1-3Galb1-4GlcNAcb1-2Mana1-3)Manb1-4GlcNAcb1-4GlcNAcb-Sp24	8976	658	7
599	GlcNAcb1-3Galb1-4GlcNAcb1-3Galb1-4GlcNAcb1-3GalNAca-Sp14	8027	177	2
548	Galb1-4GlcNAcb1-3Galb1-4GlcNAcb1-3Galb1-4GlcNAcb1-2Mana1-6(Galb1-4GlcNAcb1-3Galb1-4GlcNAcb1-3Galb1-4GlcNAcb1-2Mana1-3)Manb1-4GlcNAcb1-4GlcNAcb-Sp12	7594	1586	21
584	GlcNAcb1-3Galb1-4GlcNAcb1-3Galb1-4GlcNAcb1-6(GlcNAcb1-3Galb1-4GlcNAcb1-3Galb1-4GlcNAb1-2)Mana1-6(GlcNAcb1-3Galb1-4GlcNAcb1-3Galb1-4GlcNAcb1-2Mana1-3)Manb1-4GlcNAcb1-4(Fuca1-6)GlcNAcb-Sp24	6573	314	5
595	GlcNAcb1-3Galb1-4GlcNAcb1-6(Galb1-3)GalNAca-Sp14	6413	498	8
339	GlcNAca1-4Galb1-4GlcNAcb1-3Galb1-4GlcNAcb1-3Galb1-4GlcNAcb-Sp0	5589	187	3
592	Galb1-4GlcNAcb1-3Galb1-4GlcNAcb1-6(Galb1-4GlcNAcb1-3Galb1-4GlcNAcb1-3)GalNAca-Sp14	5379	1168	22
583	Galb1-4GlcNAcb1-3Galb1-4GlcNAcb1-6(Galb1-4GlcNAcb1-3Galb1-4GlcNAb1-2)Mana1-6(Galb1-4GlcNAcb1-3Galb1-4GlcNAcb1-2Mana1-3)Manb1-4GlcNAcb1-4(Fuca1-6)GlcNAcb-Sp24	4733	318	7
163	Galb1-4GlcNAcb1-3Galb1-4GlcNAcb1-3Galb1-4GlcNAcb-Sp0 **(type II)**	4664	164	4
590	Galb1-4GlcNAcb1-3Galb1-4GlcNAcb1-3GalNAca-Sp14	4478	268	6
337	GalNAca1-3(Fuca1-2)Galb1-4GlcNAcb1-3Galb1-4GlcNAcb1-3Galb1-4GlcNAcb-Sp0	4322	650	15
259	Neu5Aca2-3Galb1-4GlcNAcb1-3Galb1-4GlcNAcb1-3Galb1-4GlcNAcb-Sp0	4020	529	13
597	Neu5Aca2-3Galb1-4GlcNAcb1-3Galb1-4GlcNAcb1-6(Neu5Aca2-3Galb1-	3793	44	1

	4GlcNAcb1-3Galb1-4GlcNAcb1-3)GalNAca-Sp14			
609	Neu5Aca2-6Galb1-4GlcNAcb1-3Galb1-4GlcNAcb1-2Mana1-6(Neu5Aca2-6Galb1-4GlcNAcb1-3Galb1-4GlcNAcb1-2Mana1-3)Manb1-4GlcNAcb1-4GlcNAcb-Sp12	3303	276	8
573	Galb1-3GlcNAcb1-3Galb1-4GlcNAcb1-6(Galb1-3GlcNAcb1-3Galb1-4GlcNAb1-2)Mana1-6(Galb1-3GlcNAcb1-3Galb1-4GlcNAcb1-2Mana1-3)Manb1-4GlcNAcb1-4(Fuca1-6)GlcNAcb-Sp24	3287	124	4
604	Neu5Aca2-3Galb1-4GlcNAcb1-3Galb1-4GlcNAcb1-2Mana1-6(Neu5Aca2-3Galb1-4GlcNAcb1-3Galb1-4GlcNAcb1-2Mana1-3)Manb1-4GlcNAcb1-4GlcNAcb-Sp12	2830	107	4
383	Galb1-3GlcNAcb1-3Galb1-4GlcNAcb1-6(Galb1-3GlcNAcb1-3)Galb1-4Glcb-Sp0	2815	236	8
560	Galb1-4GlcNAcb1-3Galb1-4GlcNAcb1-6(Galb1-4GlcNAcb1-3Galb1-4GlcNAcb1-2)Mana1-6(Galb1-4GlcNAcb1-3Galb1-4GlcNAcb1-2Mana1-3)Mana1-4GlcNAcb1-4GlcNAc-Sp24	2282	238	10
606	Neu5Aca2-6Galb1-4GlcNAcb1-3Galb1-4GlcNAcb1-6(Neu5Aca2-6Galb1-4GlcNAcb1-3Galb1-4GlcNAcb1-3)GalNAca-Sp14	2115	399	19
607	Neu5Aca2-6Galb1-4GlcNAcb1-3Galb1-4GlcNAcb1-3Galb1-4GlcNAcb1-2Mana1-6(Neu5Aca2-6Galb1-4GlcNAcb1-3Galb1-4GlcNAcb1-3Galb1-4GlcNAcb1-2Mana1-3)Manb1-4GlcNAcb1-4GlcNAcb-Sp12	1944	345	18
593	Neu5Aca2-3Galb1-4GlcNAcb1-3Galb1-4GlcNAcb1-3GalNAca-Sp14	1917	50	3
333	Neu5Aca2-6Galb1-4GlcNAcb1-3Galb1-4GlcNAcb1-3Galb1-4GlcNAcb-Sp0	1887	171	9
71	Fuca1-2Galb1-4(Fuca1-3)GlcNAcb1-3Galb1-4(Fuca1-3)GlcNAcb1-3Galb1-4(Fuca1-3)GlcNAcb-Sp0	1859	99	5
598	Neu5Aca2-6Galb1-4GlcNAcb1-3Galb1-4GlcNAcb1-3GalNAca-Sp14	1825	80	4
546	GlcNAcb1-3Galb1-4GlcNAcb1-3Galb1-4GlcNAcb1-2Mana1-6(GlcNAcb1-3Galb1-4GlcNAcb1-3Galb1-4GlcNAcb1-2Mana1-3)Manb1-4GlcNAcb1-4GlcNAcb-Sp12	1809	364	20
544	Neu5Gca2-3Galb1-4GlcNAcb1-3Galb1-4GlcNAcb1-2Mana1-6(Neu5Gca2-3Galb1-4GlcNAcb1-3Galb1-4GlcNAcb1-2Mana1-3)Manb1-4GlcNAcb1-4GlcNAcb-Sp24	1789	40	2
185	GlcNAcb1-3Galb1-4GlcNAcb1-3Galb1-4GlcNAcb-Sp0	1611	60	4
148	Galb1-3GlcNAcb1-3Galb1-4GlcNAcb-Sp0	1134	54	5
118	Gala1-3Galb1-4Glc-Sp10	975	106	11
575	GlcNAcb1-3Galb1-4GlcNAcb1-2Mana1-6(GlcNAcb1-3Galb1-4GlcNAcb1-2Mana1-3)Manb1-4GlcNAcb1-4(Fuca1-6)GlcNAcb-Sp24	914	41	4
119	Gala1-3Galb-Sp8	897	1176	131
542	Galb1-4GlcNAcb1-3Galb1-4GlcNAcb1-2Mana1-6(Galb1-4GlcNAcb1-3Galb1-4GlcNAcb1-2Mana1-3)Manb1-4GlcNAcb1-4GlcNAcb-Sp12	800	127	16
74	Fuca1-2Galb1-4GlcNAcb1-3Galb1-4GlcNAcb-Sp0 **(H-type II)**	791	81	10
248	Neu5Aca2-3Galb1-3GlcNAcb1-3Galb1-4GlcNAcb-Sp0	598	131	22
164	Galb1-4GlcNAcb1-3Galb1-4GlcNAcb-Sp0 **(di-LacNAc, type II)**	584	27	5
366	Fuca1-4(Galb1-3)GlcNAcb1-2Mana1-6(Fuca1-4(Galb1-3)GlcNAcb1-2Mana1-3)Manb1-4GlcNAcb1-4(Fuca1-6)GlcNAcb-Sp22	563	85	15
608	Neu5Aca2-3Galb1-4GlcNAcb1-3Galb1-4GlcNAcb1-3Galb1-4GlcNAcb1-2Mana1-6(Neu5Aca2-3Galb1-4GlcNAcb1-3Galb1-4GlcNAcb1-3Galb1-4GlcNAcb1-2Mana1-3)Manb1-4GlcNAcb1-4GlcNAcb-Sp12	517	141	27
13	Glcb-Sp8	490	505	103
440	Galb1-4Galb-Sp10	464	223	48
344	GlcNAca1-4Galb1-4GlcNAcb1-3Galb1-4GlcNAcb-Sp0	455	108	24
249	Fuca1-2(6S)Galb1-4Glcb-Sp0	435	229	53
559	GlcNAcb1-3Galb1-4GlcNAcb1-6(GlcNAcb1-3Galb1-4GlcNAcb1-2)Mana1-6(GlcNAcb1-3Galb1-4GlcNAcb1-2Man a1-3)Manb1-4GlcNAcb1-4GlcNAc-Sp24	434	103	24
12	Galb-Sp8	429	201	47
475	Neu5Aca2-3Galb1-3GlcNAcb1-6(Neu5Aca2-3Galb1-4GlcNAcb1-2)Mana1-	423	607	143

	6(Neu5Aca2-3Galb1-3GlcNAcb1-2Mana1-3)Manb1-4GlcNAcb1-4GlcNAcb-Sp19			
396	Gala1-3Galb1-3(Fuca1-4)GlcNAcb1-2Mana1-6(Gala1-3Galb1-3(Fuca1-4)GlcNAcb1-2Mana1-3)Manb1-4GlcNAcb1-4GlcNAc-Sp19	418	65	16
357	KDNa2-3Galb1-4(Fuca1-3)GlcNAc-Sp0	407	243	60
541	GlcNAcb1-3Galb1-4GlcNAcb1-2Mana1-6(GlcNAcb1-3Galb1-4GlcNAcb1-2Mana1-3)Manb1-4GlcNAcb1-4GlcNAcb-Sp25	406	45	11
561	Gala1-3Galb1-4GlcNAcb1-2Mana1-6(Gala1-3Galb1-4GlcNAcb1-2Mana1-3)Manb1-4GlcNAcb1-4GlcNAc-Sp24	387	46	12
502	Fuca1-2(6S)Galb1-3(6S)GlcNAcb-Sp0	383	129	34
84	GalNAca1-3(Fuca1-2)Galb1-4(Fuca1-3)GlcNAcb-Sp0	372	99	27
456	Gala1-3(Fuca1-2)Galb1-3GlcNAcb1-2Mana1-6(Gala1-3(Fuca1-2)Galb1-3GlcNAcb1-2Mana1-3)Manb1-4GlcNAcb1-4(Fuca1-6)GlcNAcb-Sp22	372	36	10
540	GlcNAcb1-3Galb1-4GlcNAcb1-2Mana1-6(GlcNAcb1-3Galb1-4GlcNAcb1-2Mana1-3)Manb1-4GlcNAcb1-4GlcNAcb-Sp12	366	85	23
568	Galb1-3GlcNAcb1-6(Galb1-3)GalNAc-Sp14	366	74	20
166	Galb1-4GlcNAcb1-3Galb1-4Glcb-Sp8 **(LNnT, type II)**	363	86	24
380	GalNAcb1-4GlcNAcb1-2Mana1-6(GalNAcb1-4GlcNAcb1-2Mana1-3)Manb1-4GlcNAcb1-4GlcNAc-Sp12	361	76	21
596	GlcNAcb1-3Galb1-4GlcNAcb1-6(GlcNAcb1-3Galb1-4GlcNAcb1-3)GalNAca-Sp14	351	67	19
258	Neu5Aca2-3Galb1-4(Fuca1-3)GlcNAcb1-3Galb1-4GlcNAcb-Sp8	342	173	50
98	GalNAcb1-4(Fuca1-3)GlcNAcb-Sp0	329	86	26
5	GalNAca-Sp15	328	188	57
501	Fuca1-2Galb1-3(6S)GlcNAcb-Sp0	328	89	27
421	Fuca1-2Galb1-4GlcNAcb1-2Mana1-6(Fuca1-2Galb1-4GlcNAcb1-2Mana1-3)Manb1-4GlcNAcb1-4(Fuca1-6)GlcNAcb-Sp22	321	57	18
395	Gala1-3Galb1-3GlcNAcb1-2Mana1-6(Gala1-3Galb1-3GlcNAcb1-2Mana1-3)Manb1-4GlcNAcb1-4GlcNAc-Sp19	316	82	26
59	Fuca1-2Galb1-3GalNAcb1-3Gala-Sp9	305	152	50
149	Galb1-3GlcNAcb1-3Galb1-4Glcb-Sp10	303	151	50
121	Gala1-4Galb1-4GlcNAcb-Sp0	300	127	42

References

1. Ramani, S. et al. The VP8* domain of neonatal rotavirus strain G10P[11] binds to type II precursor glycans. *J Virol* **87**, 7255-64 (2013).
